# Association of plasma chemerin with all-cause and disease-specific mortality – results from a population-based study

**DOI:** 10.1038/s41366-023-01342-0

**Published:** 2023-07-25

**Authors:** Katharina Noppes, Stefan Groß, Anke Hannemann, Marcello R. P. Markus, Martin Bahls, Henry Völzke, Marcus Dörr, Matthias Nauck, Nele Friedrich, Stephanie Zylla

**Affiliations:** 1 Institute of Clinical Chemistry and Laboratory Medicine, University Medicine Greifswald, Greifswald, Germany; 2 DZHK (German Centre for Cardiovascular Research), partner site Greifswald, Greifswald, Germany; 3 Department of Internal Medicine B, University Medicine Greifswald, Greifswald, Germany; 4 DZD (German Center for Diabetes Research), site Greifswald, Greifswald, Germany; 5 Institute for Community Medicine, University Medicine Greifswald, Greifswald, Germany

**Keywords:** Endocrine system and metabolic diseases, Epidemiology

## Abstract

**Background and objectives:**

Various cross-sectional studies have observed an association between high circulating concentrations of the adipokine chemerin and an unfavorable metabolic profile. However, the prognostic value of chemerin for the risk of associated diseases and mortality was examined only in a few studies mostly using small and highly selected patient populations. We aimed to analyze the association between plasma chemerin concentrations and all-cause as well as cause-specific mortality in the general population.

**Study design and methods:**

From the Study of Health in Pomerania (SHIP), participants of two independent cohorts (SHIP-START-1 [*n* = 3037], SHIP-TREND-0 [*n* = 4193]) were followed up for 15 and 9 years (median), respectively. The association between plasma chemerin and all-cause mortality was analyzed using multivariable Cox proportional hazard regression models. Additionally, cause-specific hazards for cardiovascular disease (CVD) and cancer mortality were modeled considering competing events.

**Results:**

A total number of 507 and 208 deaths occurred during follow-up in SHIP-START-1 and SHIP-TREND-0, respectively. Multivariable regression analyses revealed a significant association between high plasma chemerin concentrations and greater overall mortality that was independent of major confounders. Each 30 ng/mL increase in chemerin was associated with a 17% higher risk of all-cause mortality (95%-confidence interval: 1.10–1.26). Cause-specific analyses further showed that the chemerin concentration was significantly associated with cancer mortality but not with CVD mortality.

**Conclusion:**

The present study detected a positive association between plasma chemerin concentrations and all-cause mortality in a large population-based study sample. Cause-specific analyses have shown that chemerin is likely to play a decisive role in cancer-related deaths. However, a direct association with cardiovascular mortality could not be established.

## Introduction

In the late 1980s and 1990s, scientific findings on adipose tissue established its role as an active endocrine organ, functioning through the production of hundreds of bioactive factors summarized as adipokines [1, 2]. Adipokines are small peptides (20–50 kDa) known to interact as signaling molecules between adipose tissue and targets in the brain, liver, immune system, and other tissues [3]. In the last two decades, investigations of their role in the regulation of important physiological functions have led to a better understanding of their relevance in various pathophysiological processes [4–6].

In this study, we will focus on chemerin, a recently discovered adipokine [7–9]. White adipose tissue is one of the main sources synthesizing chemerin [10] and various cross-sectional epidemiological studies have observed an association between high circulating chemerin concentrations and an unfavorable inflammatory and metabolic profile [11, 12]. Furthermore, associations between circulating chemerin and kidney function as well as different subclinical cardiovascular parameters were detected using cross-sectional data [13, 14]. Longitudinal studies that examined the prognostic value of chemerin concentrations for the risk, prognosis, and outcome of these conditions (e.g., heart failure, dialysis, mortality) are rare and often provided inconsistent findings [13, 15, 16]. Only a few studies have focused on the associations between circulating chemerin concentrations and mortality [13, 15, 17–23]. The majority of them provided evidence that a high chemerin concentration is associated with an increased mortality risk [17–23]. However, the comparability of existing studies is limited due to the inclusion of very small and selected patient populations. Studies that analyzed circulating chemerin concentrations and their possible link to mortality in a general population are missing so far.

Therefore, this study aimed to examine the association between plasma chemerin concentrations and mortality in two well-characterized population-based cohorts. We hypothesize that high plasma chemerin concentrations are associated with an overall as well as cause-specific higher mortality risk.

## Methods

### Study population

The Study of Health in Pomerania (SHIP) is a population-based project conducted in West Pomerania, a rural region in the northeast of Germany [24, 25]. So far, the overall research project consists of three independent cohorts (SHIP-START, SHIP-TREND, SHIP-NEXT). The cohorts are based on stratified random samples from participants aged 20–79 years that were independently drawn from the central population registry of the German Federal State of Mecklenburg-West Pomerania [24, 25]. The plasma chemerin concentration was measured in SHIP-TREND-0 as well as in the first follow-up of the SHIP-START cohort (SHIP-START-1) [25]. Therefore, the following analyses focused only on data from these two cohorts.

The baseline examinations of SHIP-START (SHIP-START-0) were conducted between 1997 and 2001 in 4307 participants from the drawn net sample of 6265 eligible individuals (69% response). A total number of 3299 participants of SHIP-START-0 participated also in the 5-year follow-up examination (SHIP-START-1), which was conducted between 2002 and 2006. SHIP-TREND is an independent cohort based on a random sample of 8826 persons who did not participate in SHIP-START. The baseline examinations of SHIP-TREND (SHIP-TREND-0) were conducted between 2008 and 2012 in 4420 of the invited participants (50% response). The study followed the principles of the Declaration of Helsinki and was approved by the ethics committee of the University of Greifswald. All participants provided written informed consent. SHIP data are publically available for scientific purposes (https://www.fvcm.med.uni-greifswald.de/dd_service/data_use_intro.php).

**Fig. 1:**
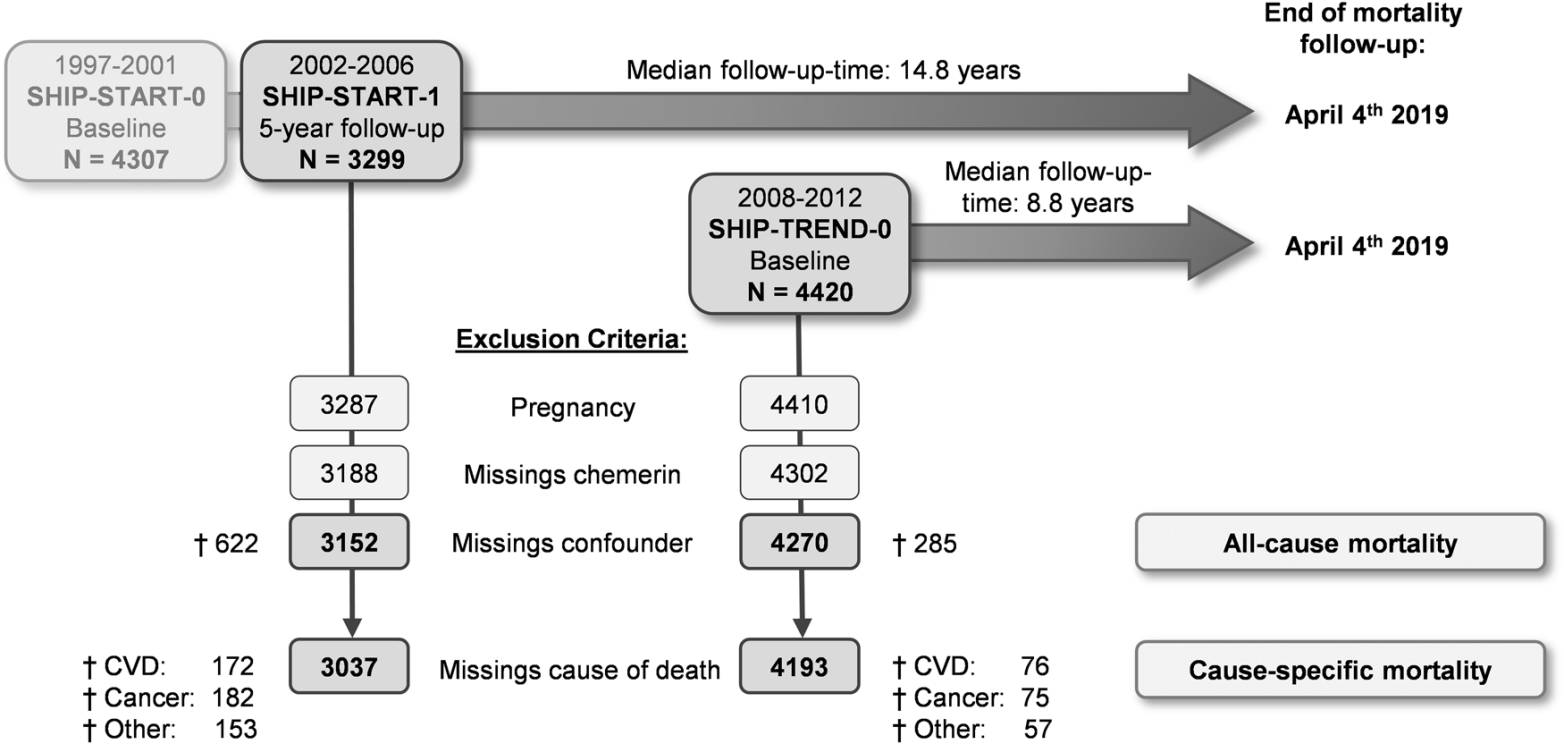
Flow diagram of the study population.

The flowchart in Fig. 1 shows the definition of the study populations for our analyses. From the 3299 SHIP-START-1 and 4420 SHIP-TREND-0 participants available for analyses, pregnant women, participants with missing data in chemerin concentrations or in any of the considered confounding variables were removed from the sample. The final study populations available for the present analyses consisted of 3152 SHIP-START-1 participants (51.8% women) aged 25 to 88 years and 4270 SHIP-TREND-0 participants (51.4% women) aged 20 to 84 years. The cause-specific analysis of mortality was done after the exclusion of participants with missing information on the underlying cause of death (SHIP-START-1: *n* = 115; SHIP-TREND-0: *n* = 77).

### Measurements

Data on age, sex, and medical histories were obtained by standardized computer-assisted personal interviews [24, 25]. The exact questions and variable definitions can be found in the data dictionaries (https://transfer.ship-med.uni-greifswald.de/FAIRequest/data-use-intro). Smoking status was categorized into current, former, or never smokers. The classification was based on participants’ self-reports. The participants were asked whether they had ever smoked daily (SHIP-START-1) or whether they had ever smoked cigarettes (SHIP-TREND-0). Mean daily alcohol consumption was calculated using beverage-specific pure ethanol-volume proportions. The waist circumference of the individuals was quantified following a standardized protocol [24]. After a 5 min resting period, blood pressure was measured three times on seated participants using a digital blood pressure monitor (HEM-705CP; Omron, Tokyo, Japan), with each reading being followed by a further resting period of three minutes [24]. The mean of the second and third measurements was taken for all further analyses.

Blood samples were collected from the cubital vein in the supine position [24]. Samples were stored at −80 °C in the Integrated Research Biobank (Liconic, Lichtenstein) of the University Medicine of Greifswald [26] and were used in accordance with its regulations. Glycated hemoglobin (HbA1c) was determined by high-performance liquid chromatography with spectrometric detection (Diamat Analyzer, Bio-Rad, Munich, Germany). Low-density lipoprotein (LDL) cholesterol was measured in SHIP-START-1 by lipoprotein electrophoresis (HELENA SAS-3 System, Helena 7 BioSciences Europe, Tyne and Wear, UK) and in SHIP-TREND-0 by photometry (Dimension VISTA 1500, Siemens Healthineers, Erlangen, Germany). Serum cystatin C concentrations were assessed using a nephelometric assay (SHIP-START-1: BN^TM^ II, Siemens Healthcare Diagnostics, Eschborn, Germany; SHIP-TREND-0: Dimension Vista, Siemens Healthcare Diagnostics, Eschborn, Germany). The Chronic Kidney Disease Epidemiology Collaboration (CKD-EPI) equation [27] was used to calculate the estimated glomerular filtration rate (eGFR) based on cystatin C concentrations. Plasma chemerin concentrations were measured using a commercially available enzyme-linked immunosorbent assay (ELISA) technique (Mediagnost, Reutlingen, Germany). The inter-assay coefficients of variation were 9.5% and 8.4% (SHIP-START-1) and 5.8% and 5.5% (SHIP-TREND-0) for low and high concentrations of the control material, respectively.

### Follow-up of vital status

From the time of enrollment into the study until April 4th, 2019, the information on vital status was acquired at regular intervals from the central registration office of the federal state of Mecklenburg-West Pomerania. This central registration office can only provide the vital status for study participants who were registered in the state of Mecklenburg-West Pomerania at the time of query or time of death. For those participants who have moved away, it is more difficult to record their vital status. In these cases, information about death can only be recorded if the relatives of a deceased person report back the time of death. If a participant’s vital status is unknown, that participant will be assumed to have dropped out of the study and is treated as censored at the time of the last contact (*n* = 1).

Death certificates were requested for all known deaths from the local health authority of the residence of death. A certified nosologist coded the underlying cause of death according to the International Classification of Diseases, 10th revision (ICD-10). Additionally, two internists (M.D., H.V.) independently validated the underlying cause of death and performed a joint reading in cases of disagreement. The age at death or censoring (in years), follow-up time (in days), and ICD-10 coded major causes of death were available for mortality analysis. Missing or unseen death certificates and uncertainties in the coding of the underlying cause may explain missing values in the cause of death.

Participants were included in the analyses until the time of death or censoring (latest vital status assessment or latest contact). The median duration of follow-up was 14.8 years for SHIP-START-1 participants (25th quartile: 14.0 years; 75th quartile: 15.6 years) and 8.8 years for SHIP-TREND-0 participants (25th quartile: 7.9 years; 75th quartile: 9.5 years). Altogether, we observed 42522 person-years of follow-up in SHIP-START-1 and 36026 person-years of follow-up in SHIP-TREND-0.

Participants’ age was used as the time scale in the present analyses (unit: years). The participants entered the analysis at their age at the examination of SHIP-START-1 or SHIP-TREND-0 (left-truncation) and exited at their event or censoring age. With age as the primary time scale using left-truncation on age at entry, it was not necessary to further adjust the regression models for age at entry in the study [28].

### Statistical analyses

For the statistical analyses, data from both study cohorts were combined (*N* = 7422). The overall study population was divided into three groups according to the tertiles of chemerin. Continuous data were presented as median (25th quartile; 75th quartile). Nominal data were given as percentages. Crude, as well as age-standardized mortality rates, were calculated separately for participants with low, mid, and high concentrations of chemerin (per 1000 person-years). The overall population was used as the standard population for direct age standardization of the mortality rate. Furthermore, sex-adjusted Cox proportional hazards regression models with restricted cubic splines for chemerin (three knots located at the 5th, 50th, 95th percentile) were estimated to detect a possible non-linear relationship between chemerin and the log hazard function for all-cause, CVD, and cancer mortality. Spline functions were calculated for the study population without missing values for the cause of death (*N* = 7230) as well as for participants aged < 60 years (*n* = 4662), 60−69 years (*n* = 1446), and ≥ 70 years (*n* = 1122), separately. Participants’ age was used as time scale and the study cohort was included as a stratification variable in these regression models.

Based on these results, we decided to consider chemerin as a continuous parameter (per 30 ng/mL [standard deviation]) in subsequent Cox proportional hazards regression models analyzing the association between circulating chemerin and mortality. In these regression models, participants’ age was used as time scale and the following variables were included as confounding factors: sex, waist circumference, HbA1c, smoking status, and eGFR. The selection of these covariables was based on a directed acyclic graph (DAG) generated by all authors of this project (Supplementary Fig. S1). As we assumed that the two study cohorts have different baseline hazard functions, we included the study cohort as a stratification variable in the regression models. Effect estimates were presented as hazard ratios together with the 95%-confidence interval. The proportional hazard assumption was visually inspected and confirmed by plotting scaled Schoenfeld residuals against the function of time for chemerin as exposure. Sex-specific analyses were not necessary as interaction terms of chemerin with sex revealed no significant effect modification.

We calculated cause-specific hazard models for the two most common causes of death (cardiovascular disease [CVD], cancer). The results for the group of other causes of death are presented here but not discussed because the group of these participants was too unspecific. Cause-specific hazard regressions were modeled for each event separately by using the cause-specific hazard function [29]. Thus, those participants who experienced a competing event were treated as being censored at the time of occurrence of the competing event [29].

To check the stability of the results, we repeated all regression analyses after additional adjustments for systolic blood pressure and LDL-cholesterol as well as in the two study cohorts separately. Statistical significance was assumed at a *p*-value < 0.05.

## Results

### Clinical and biometrical data of the study population

Table 1 provides an overview of the descriptive statistics of the study population separated by chemerin concentration. On average, participants with higher chemerin concentrations were more likely to be female, were older, had a higher waist circumference, and had a less favorable metabolic profile than participants with lower chemerin concentrations. A comparison of the descriptive statistics for the two study cohorts is shown in Supplemental Table S1.

**Table 1:** Descriptive statistics of the study population separated by plasma chemerin concentrations.

	low chemerin	mid chemerin	high chemerin
	18 – < 86 ng/mL	86 – < 109 ng/mL	109 – < 329 ng/mL
	(*n* = 2451)	(*n* = 2444)	(*n* = 2527)
Age (years)	45 (35; 57)	53 (41; 64)	61 (50; 71)
Sex (% female)	45.0	51.2	58.5
Smoking (%)			
never smokers	37.4	36.3	41.9
former smokers	32.2	36.9	35.4
current smokers	30.4	26.8	22.7
Alcohol consumption (g/day)	5.1 (1.4; 13.4)	3.9 (1.0; 11.2)	2.2 (0.4; 7.8)
Waist circumference (cm)	84.8 (75.3; 94.0)	91.5 (81.6; 101.0)	97.3 (88.3; 106.3)
HbA1c (%)	5.1 (4.7; 5.4)	5.3 (4.9; 5.6)	5.5 (5.1; 6.0)
Systolic blood pressure (mmHg)	125 (113; 137)	129 (116; 141)	132 (121; 145)
LDL-cholesterol (mmol/L)	3.2 (2.6; 3.8)	3.4 (2.8; 4.1)	3.5 (2.9; 4.2)
eGFR (mL/min per 1.73 m²)	115.5 (105.8; 123.7)	108.0 (95.5; 117.1)	94.4 (72.1; 108.8)
Chemerin (ng/mL)	74.3 (65.9; 80.4)	96.7 (91.2; 102.6)	126.9 (116.7; 143.4)
Person-years	27214	26148	25186
Number of deaths (all-cause)	161	229	517
Crude mortality rate*	5.92	8.76	20.53
Age-standardized mortality rate*	9.66	9.29	13.68
Number of deaths (cause of death)			
CVD	33	62	153
Cancer	52	64	141
Other	38	49	123
Missing	38	54	100

The overall study population was divided into three groups according to the tertiles of the plasma chemerin concentration. Continuous data are presented as median (25th quartile; 75th quartile); nominal data are given as percentages. *All-cause mortality rates were calculated per 1000 person-years. The overall population was used as the standard population for direct age standardization of the mortality rates. *HbA1c* Glycated hemoglobin, *LDL* Low-density lipoprotein, *eGFR* Estimated glomerular filtration rate, *CVD* Cardiovascular disease.

From both SHIP cohorts, 907 participants died during 78549 person-years of follow-up. In total, 248 and 257 of these deaths were due to CVD and cancer, respectively. The most common causes of death were malignant neoplasms of the bronchus and lung (ICD-10: C34, *n* = 49), acute myocardial infarction (ICD-10: I21, *n* = 46), heart failure (ICD-10: I50, *n* = 42), chronic ischaemic heart disease (ICD-10: I25, *n* = 33), and cardiac arrest (ICD-10: I46, *n* = 24). Supplementary Table S2 provides a complete overview of the number of deaths broken down by CVD, cancer, and other causes and by groups of ICD-10 diagnosis codes.

### Vital status depending on chemerin concentration

The crude and age-standardized all-cause mortality rates depending on tertiles of chemerin are presented in Table 1. It can be observed that the crude mortality rate is higher with increasing chemerin concentration. However, the age-standardized mortality rate shows that the differences in mortality rate between participants with low and intermediate chemerin concentrations are negligible, but the age-standardized mortality rate of participants with chemerin concentrations above the highest tertile was higher than that of participants with lower or intermediate chemerin concentrations.

**Fig. 2:**
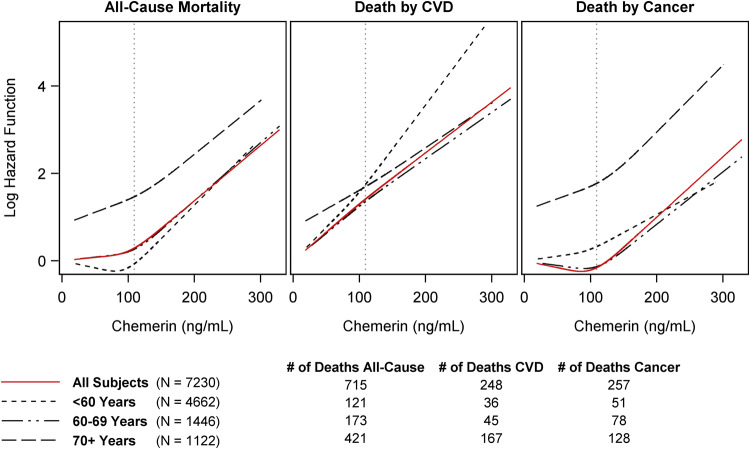
Predicted log hazard function for all-cause, cardiovascular diseases (CVD), and cancer mortality in relation to plasma chemerin concentration. Results are based on sex-adjusted Cox proportional hazard regression models with restricted cubic splines for chemerin (three knots located at the 5th, 50th, 95th percentile). The study cohort was included as a stratification variable and participants’ age was used as time scale (unit: years). Spline functions and regression models were calculated for the whole study population as well as for participants aged < 60 years, 60–69 years, and ≥ 70 years, separately. Cause-specific hazards were modeled for each event separately and the respective competing events were considered as censored cases (cause-specific hazard function). The dotted lines colored in grey represent the highest tertile (109 ng/mL) of chemerin concentration in the overall population.

To determine whether the observed effect between chemerin and mortality risk could be fully explained by the influence of age and sex, we predicted the sex-adjusted log hazard of all-cause, CVD, and cancer mortality as a function of chemerin for the entire study population (without missing values in the cause of death, *n* = 7230) as well as for the age groups < 60 years (*n* = 4662), 60–69 years (*n* = 1446), and ≥ 70 years (*n* = 1122). Participants’ age was used as time scale. The results, presented in Fig. 2, show that the shape of the log hazard depending on chemerin was comparable for the different age groups and causes of death. Thus, the observed relation between chemerin and mortality cannot be explained by age and sex alone. Furthermore, the predicted log hazard argued for a positive linear relation between chemerin and the risk of mortality. Therefore, we decided to consider chemerin continuously in the subsequent regression analyses.

### Association of chemerin with all-cause and cause-specific mortality

Adjusted analyses using multivariable Cox proportional hazards models demonstrated that each increase in chemerin by 30 ng/mL was associated with a 17% higher risk of all-cause mortality (Table 2). Cause-specific hazard ratios for CVD and cancer as causes of death were calculated using competing risk regression in a subpopulation without missing values for the cause of death (Table 2). We observed that the chemerin concentration was significantly associated with cancer mortality but not with mortality due to CVD or other causes.

**Table 2:** Association between plasma chemerin concentration and all-cause as well as cause-specific mortality based on Cox proportional hazards regression models.

Cause of death	# deaths/n	HR (95%-CI)	*p*-value
**Analysis of all-cause mortality**			
All-cause	907/7422	1.17 (1.10; 1.26)	4.488E-06
**Analysis of cause-specific mortality (considering competing risks)**			
All-cause	715/7230	1.18 (1.10; 1.28)	1.822E-05
CVD	248/7230	1.13 (0.99; 1.28)	6.394E-02
Cancer	257/7230	1.28 (1.13; 1.46)	1.242E-04
Other	210/7230	1.14 (0.99; 1.32)	6.817E-02

The models were adjusted for sex, waist circumference, glycated hemoglobin, smoking status, and estimated glomerular filtration rate. The study cohort was included as a stratification variable. Participants’ age was used as time scale (unit: years). Chemerin was modeled as a continuous parameter (per 30 ng/mL [standard deviation]). Effect estimates were presented as hazard ratio (HR) together with its 95%-confidence interval (CI). Cause-specific hazards were modeled for each event separately and the respective competing events were considered as censored cases (cause-specific hazard function). *CVD* Cardiovascular disease.

Additional adjustments for systolic blood pressure and LDL-cholesterol did not change the regression results (Supplementary Table S3). The results remained comparatively stable even when data from both study cohorts were analyzed separately (Supplementary Table S4). However, the association between chemerin and CVD as the cause of death was borderline significant when SHIP-START-1 was considered alone.

## Discussion

In the present study, we used population-based data to analyze the association of plasma chemerin concentrations with all-cause and disease-specific mortality. The results showed a significant association between high chemerin concentrations and a greater overall mortality risk. After adjusting for relevant confounders, we observed that each 30 ng/mL increase in chemerin was associated with a 17% higher risk of all-cause mortality. Cause-specific analyses further showed that the chemerin concentration was significantly associated with cancer mortality but not with mortality due to CVD or other causes of death.

### Chemerin and all-cause mortality

Very few studies have investigated the associations between circulating chemerin concentrations and all-cause mortality [13, 15, 17–23]. The examinations were based exclusively on highly selected patient populations with small sample sizes [13, 15, 17–23]. Furthermore, most studies investigated the risk of all-cause mortality in combination with other adverse events or in subordinate analyses [15, 18, 20–23]. Together, only four studies were identified that focused within their analyses on all-cause mortality as a primary endpoint [13, 17–19].

Yamamoto et al. [13] were the first who studied the association between chemerin and all-cause mortality. They analyzed the mortality rate among 282 patients with kidney failure over a 5-year follow-up period (63 patients died). Using Cox regression models adjusted for age, sex, and C-reactive protein, they observed that a low chemerin concentration ( < 128.6 ng/mL) was associated with higher mortality [13]. Thus, it appears that elevated chemerin may have a protective effect on the survival of dialysis patients [13]. In contrast, a more recent study [19], which also observed patients with chronic renal failure (*n* = 98), has shown the opposite relationship. Here, chemerin concentrations were significantly higher in patients who died during the 1-year follow-up period (*n* = 19) than in those who survived [19]. Er et al. [18] analyzed data from 481 patients with confirmed coronary artery disease who were followed up for 1022 days ( ± 320 days) and of whom 27 died during the study period. Consistent with our findings and those presented by Liu et al. [19], the authors have observed that a higher chemerin concentration ( > 163.8 ng/mL) was an independent predictor of an increased all-cause mortality [18]. Similar to our analyses, Cox regression models adjusted for age, sex, body mass index, smoking status, diabetes, hypertension, and dyslipidemia were applied to analyze the relationship. Finally, also Xu et al. [17] found that a high chemerin concentration was associated with a higher risk of all-cause mortality by examining data from 189 patients with non-small cell lung cancer during a 5-year follow-up period (the number of observed deaths was not published). Besides age and sex, the Cox model used in this study was adjusted for several cancer progression factors [17].

Four other studies investigated the relationship between chemerin and mortality in secondary analyses [20–23]. Chen et al. [20], Zhou et al. [21], and Wang et al. [23] focused their analyses on the association between chemerin and the occurrence of major cardiac events in patients with CVD. Zhang et al. [22] studied the prognosis of patients with gastric cancer. Consistent with our findings, all of the aforementioned studies observed a positive association between high chemerin concentrations and all-cause mortality [20–23].

Overall, the presented findings are quite difficult to compare with previous studies [13, 15, 17–23], because they were derived from very different populations that vary in size, health status, observed deaths, and follow-up times. Furthermore, the statistical methods and adjustment sets used to analyze the relationship between chemerin and mortality differed greatly. Additionally, each study defined a ‘high chemerin concentration’ by using its cutoff values. However, this is unavoidable as chemerin concentrations were measured using ELISA kits from different manufacturers. Nevertheless, our findings support the great majority of existing results reflecting a positive association between high chemerin concentrations and an increased all-cause mortality risk.

### Chemerin and cancer

When looking at cause-specific mortality, our data reflects a strong relationship between high plasma chemerin concentrations and cancer-related death. A recent meta-analysis [30] has shown that circulating chemerin levels were significantly higher in cancer patients than in healthy controls. However, it is not clear whether the observed associations are causal or whether chemerin has tumor-promoting or tumor-inhibiting properties. A study on 36 patients with gastric cancer has shown that serum chemerin concentrations were positively associated with advanced clinical stages of the disease, potentially predicting a patient’s survival outcome [31]. In line with this, it was demonstrated that a high chemerin expression in breast cancer tissue is associated with a poor prognostic outcome, tumor size and grading as well as metastasis [32]. In contrast, a study analyzing data from patients with hepatocellular carcinoma observed that a low chemerin concentration reflected poor clinical overall survival and a higher recurrence rate [33]. Treeck et al. [34] summarized these and other findings in a meta-analysis, highlighting the difference in survival outcome and prognosis depending on the expression of chemerin and its receptors by tumor cells and tissues. A high expression of chemerin in breast cancer, ovarian cancer, and gastric cancer seems to harm overall survival or relapse-free survival [34]. In contrast, a high expression of the chemerin receptors in tumor tissue of breast cancer, ovarian cancer, and small-cell lung cancer seems to have a beneficial effect on survival [34]. Therefore, chemerin and its receptors seem to play very divergent roles in the development and regulation of different types of cancer. This might be explained by the bidirectional role of chemerin, exerting pro-inflammatory and anti-inflammatory effects depending on its concentration, proteolytic processing, systemic vs. local circulation, and the tumor type [31, 32, 35–37]. For example, an in vitro experiment has shown that chemerin can have tumorigenesis effects on gastric cancer cells by activating matrix metalloproteinase and mitogen-activated protein kinase signaling [31]. In contrast, antitumorigenic effects have been reported for instance in patients with non-small cell lung cancer by recruiting natural killer cells [35].

Taken together, elevated circulating chemerin concentrations have been found to indicate several malignant or benign tumors and may be seen as a prognostic marker for the mortality risk of these patients. Nevertheless, disease progression and survival seem to depend strongly on the expression of chemerin and its receptors by specific tumor tissues. Thus, it is conceivable that chemerin may only be suitable for a few specific types of cancer.

### Chemerin and CVD

The present analyses showed no significant association between an increase in plasma chemerin concentrations and CVD-related deaths. Altogether, previous studies have been able to establish a link between chemerin and CVD. In patient groups with coronary artery disease, researchers found that chemerin was significantly higher compared to healthy controls [23, 38, 39]. Furthermore, it was observed that elevated chemerin concentrations are associated with endothelial dysfunction and increased arterial stiffness in hypertensive patients [40]. The results of studies investigating the associations between chemerin and different subclinical markers of atherosclerosis (e.g., carotid intima-media thickness, ankle-brachial index) are contradictory [14, 41]. The aforementioned studies are mostly based on cross-sectional data [14, 38–41]. Longitudinal examinations analyzing the causal relationship between chemerin and CVD are rare [16, 20, 21, 23, 42]. In 2190 participants from the EPIC-Potsdam cohort [16], 214 patients with dilated cardiomyopathy [20], 834 patients with chronic heart failure [21], and 77 patients with proven coronary artery disease [23], circulating chemerin was positively associated with the risk of major cardiac events. Similarly, a study analyzing data from 495 patients undergoing coronary angiography has shown that patients in the high chemerin tertile group were more often affected by cardiovascular events than patients with median or low chemerin concentrations [42]. In contrast to the present study, cardiac mortality was always analyzed in combination with other cardiac events (e.g., stroke, myocardial infarction, heart failure) [20, 21, 23, 42] but not as a single outcome considering competing events, as we did. Additionally, the studies analyzing cardiac mortality [20, 21, 23, 42] focused exclusively on patients with known and presumably treated CVD. Thus, variations in statistical methods and sample selection might explain the detected difference in our results.

In general, previous studies have shown a relationship between circulating chemerin concentrations and CVD. It seems that chemerin has proinflammatory activities in the genesis of atherosclerotic lesions [43]. However, the underlying pathophysiological processes are multifarious and remain to be fully understood. An in vitro study on cultured cardiomyocytes has shown that chemerin induced apoptosis in a time- and dose-dependent manner [44]. Furthermore, it was noticed that chemerin expression is induced through TNF alpha, an inflammation-promoting molecule [44]. Nevertheless, the predictive character of chemerin concentrations for CVD, and especially cardiovascular mortality itself, has still to be confirmed in further prospective and experimental studies, as existing data is still quite sparse. Based on the presented results, we assume that cardiac mortality itself is determined mainly by classical risk factors (e.g., hypertension, diabetes mellitus, smoking, or physical inactivity) rather than by circulating chemerin. However, the borderline significant results could also provide evidence that the associations are only apparent in specific CVD.

### Strengths and limitations

This is the first study analyzing the association between circulating chemerin concentrations and all-cause as well as disease-specific mortality in a general population. The size of our study population and the prospective character of the study, collecting data over a decade, must be emphasized. Furthermore, the present examination is characterized by a highly standardized data collection, an accurate assessment of causes of death, and an appropriate consideration of metabolic and cardiovascular risk factors. However, due to the actual procedure of reviewing and coding death certificates in SHIP, it was not possible to consider contributing causes of death in the present analyses. Furthermore, the slightly different questions on smoking behavior in the two study cohorts are to be criticized. A more precise definition of smoking behavior would have further strengthened our results. Future studies on the association between chemerin and mortality would further benefit from the analysis of expression levels of chemerin and its receptors in different tissues and further stratification of types of cancer and CVD.

## Conclusion

The present study has shown that a high plasma chemerin concentration is associated with an increased risk of all-cause mortality in the general population. Cause-specific analyses have further indicated that the chemerin concentration was significantly associated with cancer mortality but not with mortality due to CVD or other causes of death. Therefore, it seems that cancer mortality in particular is driven by circulating chemerin concentration. Overall, the presented findings highlight the importance of further research to understand the effect of chemerin expression on survival. A more complex knowledge of its proteolytic processing and its different receptors in pro- or anti-inflammatory cascades is therefore urgently required.

### Supplementary information

Supplemental Material

## Data Availability

The data that support the findings of this study are available from the “FVCM Transfer Unit for Data and Biomaterials” (https://www.fvcm.med.uni-greifswald.de/dd_service/data_use_intro.php) but restrictions apply to the availability of these data, which were used under license for the current study, and so are not publicly available. Data are however available from the authors upon reasonable request and with permission of “FVCM Transfer Unit for Data and Biomaterials”.

## Code availability

Statistical analyses were performed using SAS 9.4 (SAS Institute Inc., Cary, North Carolina, USA). The computer code for data cleaning and analysis can be viewed on request.
