# Supplementary material for: Association of plasma chemerin with all-cause and disease-specific mortality – results from a population-based study
Source: Int J Obes (Lond). 2023 Jul 25;47(10):956–62. doi: 10.1038/s41366-023-01342-0 (PMC10511313; doi:10.1038/s41366-023-01342-0)
Supplement: Supplementary file 1 — Supplemental Material [file 41366_2023_1342_MOESM1_ESM.docx]

**Supplemental Material**

**Association of Plasma Chemerin with All-Cause and Disease-Specific Mortality – Results from a Population-Based Study**

Katharina Noppes^1^, Stefan Groß^2,3^, Anke Hannemann^1,2^, Marcello R. P. Markus^2,3,4^, Martin Bahls^2,3^, Henry Völzke^2,4,5^, Marcus Dörr^2,3^, Matthias Nauck^1,2^, Nele Friedrich^1,2^, Stephanie Zylla^1,2^

1. Institute of Clinical Chemistry and Laboratory Medicine, University Medicine Greifswald, Germany
2. DZHK (German Center for Cardiovascular Research), partner site Greifswald, Germany
3. Department of Internal Medicine B, University Medicine Greifswald, Germany.
4. DZD (German Center for Diabetes Research), site Greifswald, Germany
5. Institute for Community Medicine, University Medicine Greifswald, Germany

*Address for correspondence:*

Stephanie Zylla

Institute of Clinical Chemistry and Laboratory Medicine

University Medicine Greifswald

Ferdinand-Sauerbruch-Straße NK

D-17475 Greifswald

Germany

Phone: +49 - 3834 - 865524

FAX: +49 - 3834 - 865502

E-mail: stephanie.zylla@med.uni-greifswald.de

**Figure S1:** Directed acyclic graph (DAG) showing causal assumptions on the effect of chemerin (exposure) on mortality (outcome).


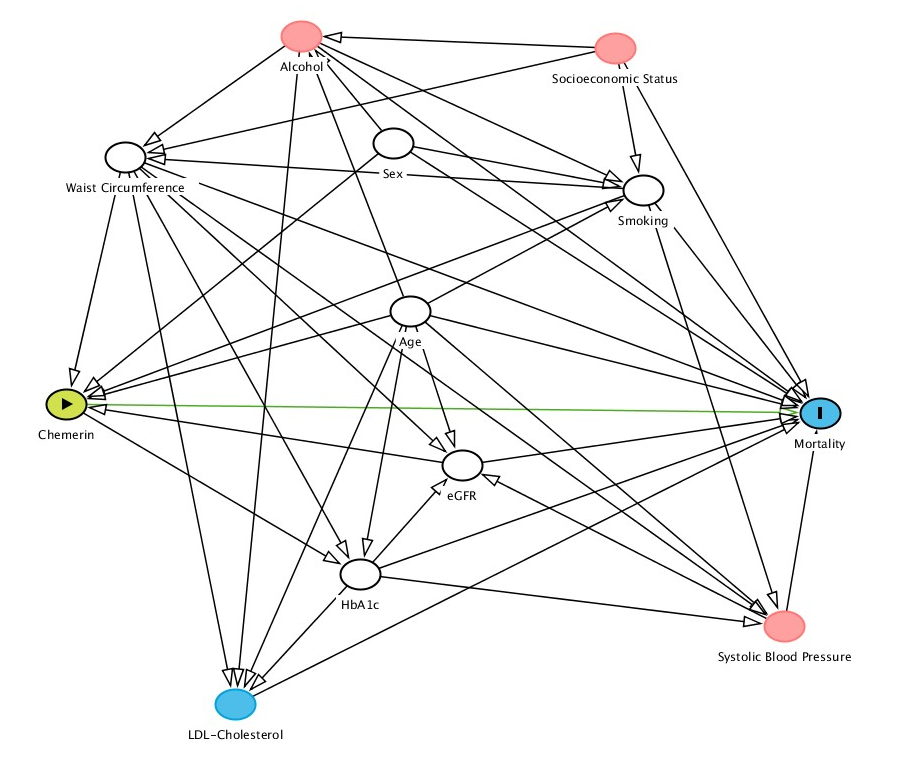


The DAG was built using DAGitty v3.0 software (available at <http://dagitty.net/development/dags>). The selection of the variables was based on biological mechanisms or evidence from previously published data. The minimal sufficient adjustment set for estimating the total effect of chemerin on mortality included age, sex, HbA1c, smoking, waist circumference, and eGFR (white circles). A green circle with a black outline: exposure; a blue circle with a black outline: outcome; a white circle: adjusted confounder, a blue circle: mediator; a red circle: confounders that are already controlled by the adjustment made. HbA1c = glycated hemoglobin; LDL = low-density lipoprotein; eGFR = estimated glomerular filtration rate.

**Table S1:** Descriptive statistics of the study population separated by study cohort.

|  | **SHIP-START-1** | **SHIP-TREND-0** |
| --- | --- | --- |
|  | **(n = 3152)** | **(n = 4270)** |
| Age (years) | 54 (42; 66) | 53 (40; 64) |
| Sex (% female) | 51.8 | 51.4 |
| Smoking (%) |  |  |
| never smokers | 41.7 | 36.2 |
| former smokers | 32.1 | 36.9 |
| current smokers | 26.2 | 26.9 |
| Alcohol consumption (g/day) | 3.9 (1.0; 11.6) | 3.4 (0.7; 10.5) |
| Waist circumference (cm) | 92.2 (82.5; 102.0) | 90.5 (80.2; 101.0) |
| HbA1c (%) | 5.3 (4.9; 5.7) | 5.3 (4.9; 5.7) |
| Systolic blood pressure (mmHg) | 131 (119; 144) | 127 (115; 140) |
| LDL-cholesterol (mmol/L) | 3.4 (2.8; 4.2) | 3.3 (2.7; 4.0) |
| eGFR (mL/min per 1.73m²) | 100.0 (80.3; 112.3) | 111.3 (98.9; 121.3) |
| Chemerin (ng/mL) | 95.6 (78.7; 116.3) | 98.0 (82.2; 117.9) |
| Person-years | 42522 | 36026 |
| Number of deaths (all-cause) | 622 | 285 |
| Number of deaths (cause of death) |  |  |
| CVD | 172 | 76 |
| cancer | 182 | 75 |
| other | 153 | 57 |
| missing | 115 | 77 |

Continuous data are presented as median (25th quartile; 75th quartile); nominal data are given as percentages. HbA1c = glycated hemoglobin; LDL = low-density lipoprotein; eGFR = estimated glomerular filtration rate; CVD = cardiovascular diseases.

**Table S2:** Overview of the number of causes of death grouped by cardiovascular diseases, cancer, and other causes and by International Classification of Diseases (ICD-10, 10th revision) diagnosis code.

| **Causes of Death** | | **ICD-10**  **Code Groups** | **# of Cases** |
| --- | --- | --- | --- |
| **CARDIOVASCULAR DISEASES** | |  | **248** |
|  | Other forms of heart disease | I30-I52 | 88 |
|  | Ischaemic heart diseases | I20-I25 | 80 |
|  | Cerebrovascular diseases | I60-I69 | 49 |
|  | Diseases of arteries, arterioles, and capillaries | I70-I79 | 15 |
|  | Pulmonary heart diseases | I26-I28 | 9 |
|  | Hypertensive diseases | I10-I15 | 6 |
|  | Ill-defined and unknown causes of mortality | R95-R99 | 1 |
| **CANCER/NEOPLASMS** | |  | **257** |
|  | Digestive organs | C15-C26 | 75 |
|  | Respiratory and intrathoracic organs | C30-C39 | 50 |
|  | Urinary tract | C64-C68 | 26 |
|  | Lymphoid, haematopoietic and related tissue | C81-C96 | 24 |
|  | Male genital organs | C60-C63 | 23 |
|  | Breast | C50-C50 | 16 |
|  | Secondary and unspecified sites | C76-C80 | 12 |
|  | Female genital organs | C51-C58 | 11 |
|  | Skin | C43-C44 | 9 |
|  | Eye, brain, and other parts of the central nervous system | C69-C72 | 4 |
|  | Mesothelial and soft tissue | C45-C49 | 3 |
|  | Thyroid and other endocrine glands | C73-C75 | 2 |
|  | Independent (primary) multiple sites | C97-C97 | 1 |
|  | Lip, oral cavity, and pharynx | C00-C14 | 1 |
| **OTHER** | |  | **210** |
|  | Diseases of the respiratory system | J00-J99 | 50 |
|  | Mental and behavioral disorders | F00-F99 | 34 |
|  | Diseases of the digestive system | K00-K93 | 32 |
|  | Symptoms, signs, abnormal clinical/laboratory findings, not elsewhere classified | R00-R99 | 27 |
|  | Injury, poisoning, and certain other consequences of external causes | S00-T98 | 20 |
|  | Diseases of the nervous system | G00-G99 | 16 |
|  | Diseases of the genitourinary system | N00-N99 | 9 |
|  | Certain infectious and parasitic diseases | A00-B99 | 7 |
|  | Endocrine, nutritional, and metabolic diseases | E00-E90 | 6 |
|  | Neoplasms of uncertain or unknown behavior | D37-D48 | 3 |
|  | Diseases of the blood and blood-forming organs | D50-D90 | 2 |
|  | Diseases of the musculoskeletal system | M00-M99 | 2 |
|  | External causes of morbidity and mortality | V01-Y84 | 2 |

**Table S3:** Association between plasma chemerin concentration and all-cause as well as cause-specific mortality based on Cox proportional hazards regression models after additional adjustment for systolic blood pressure and LDL-cholesterol.

| **Cause of death** |  | **# deaths/n** | **HR (95%-CI)** | **p-value** |
| --- | --- | --- | --- | --- |
| **Analysis of all-cause mortality** | | |  |  |
| All-cause |  | 904/7409 | 1.17 (1.10; 1.26) | 4.583E-06 |
| **Analysis of cause-specific mortality (considering competing risks)** | | | | |
| All-cause |  | 712/7217 | 1.18 (1.10; 1.28) | 1.680E-05 |
| CVD |  | 245/7217 | 1.13 (0.99; 1.28) | 6.654E-02 |
| Cancer |  | 257/7217 | 1.29 (1.13; 1.46) | 9.827E-05 |
| Other |  | 210/7217 | 1.14 (0.99; 1.32) | 6.992E-02 |

The models were adjusted for sex, waist circumference, glycated hemoglobin, smoking status, estimated glomerular filtration rate, systolic blood pressure, and LDL-cholesterol. The study cohort was included as a stratification variable. Participants’ age was used as time scale (unit: years). Chemerin was modeled as a continuous parameter (per 30 ng/mL [standard deviation]). Effect estimates were presented as hazard ratio (HR) together with its 95%-confidence interval (CI). Cause-specific hazards were modeled for each event separately and the respective competing events were considered as censored cases (cause-specific hazard function). CVD = cardiovascular disease.

**Table S4:** Association between plasma chemerin concentration and all-cause as well as cause-specific mortality based on Cox proportional hazards regression models for both study cohorts separately.

|  |  | **Cause of death** |  | **# deaths/n** | **HR (95%-CI)** | **p-value** |
| --- | --- | --- | --- | --- | --- | --- |
| **Analysis of all-cause mortality** | | | | | | |
|  | SHIP-START-1 | All-cause |  | 622/3152 | 1.18 (1.09; 1.28) | 8.444E-05 |
|  | SHIP-TREND-0 | All-cause |  | 285/4270 | 1.20 (1.05; 1.36) | 6.886E-03 |
| **Analysis of cause-specific mortality (considering competing risks)** | | | | | |  |
|  | SHIP-START-1 | All-cause |  | 507/3037 | 1.17 (1.07; 1.28) | 5.772E-04 |
|  |  | CVD |  | 172/3037 | 1.18 (1.01; 1.37) | 3.482E-02 |
|  |  | Cancer |  | 182/3037 | 1.20 (1.03; 1.40) | 1.861E-02 |
|  |  | Other |  | 153/3037 | 1.14 (0.97; 1.34) | 1.235E-01 |
|  | SHIP-TREND-0 | All-cause |  | 208/4193 | 1.25 (1.07; 1.45) | 4.110E-03 |
|  |  | CVD |  | 76/4193 | 1.06 (0.82; 1.37) | 6.392E-01 |
|  |  | Cancer |  | 75/4193 | 1.56 (1.23; 1.97) | 2.197E-04 |
|  |  | Other |  | 57/4193 | 1.14 (0.83; 1.55) | 4.203E-01 |

The models were adjusted for sex, waist circumference, glycated hemoglobin, smoking status, and estimated glomerular filtration rate. Participants’ age was used as time scale (unit: years). Chemerin was modeled as a continuous parameter (per 30 ng/mL [standard deviation]). Effect estimates were presented as hazard ratio (HR) together with its 95%-confidence interval (CI). Cause-specific hazards were modeled for each event separately and the respective competing events were considered as censored cases (cause-specific hazard function). CVD = cardiovascular disease.
